# Ursolic acid-enriched kudingcha extract enhances the antitumor activity of bacteria-mediated cancer immunotherapy

**DOI:** 10.1186/s12906-022-03612-2

**Published:** 2022-05-04

**Authors:** Haixia Xu, Linghua Piao, Xiande Liu, Sheng-nan Jiang

**Affiliations:** 1 School of Life Sciences, Hainan University, No. 58 Renmin Avenue, Haikou, 570228 China; 2 Department of Physiology, Hainan Medical University, Haikou, China; 3 Department of Nuclear Medicine, Central South University, Xiangya School of Medicine, Affiliated Haikou Hospital, No. 43 Renmin Avenue, Haikou, 570208 China

**Keywords:** BCI, Ursolic acid-enriched KDCE, Antiangiogenesis, VEGFR2

## Abstract

**Background:**

Bacteria-mediated cancer immunotherapy (BCI) robustly stimulates the immune system and represses angiogenesis, but tumor recurrence and metastasis commonly occur after BCI. The natural product *Ilex kudingcha* C. J Tseng enriched with ursolic acid has anti-cancer activity and could potentially augment the therapeutic effects of BCI. The objective of the present study was to determine potential additive effects of these modalities.

**Methods:**

We investigated the anti-cancer activity of KDCE (Kudingcha extract) combined with S.t△ppGpp in the mice colon cancer models.

**Results:**

In the present study, KDCE combined with S.t△ppGpp BCI improved antitumor therapeutic efficacy compared to S.t△ppGpp or KDCE alone. KDCE did not prolong bacterial tumor-colonizing time, but enhanced the antiangiogenic effect of S.t△ppGpp by downregulatingVEGFR2. We speculated that KDCE-induced VEGFR2 downregulation is associated with FAK/MMP9/STAT3 axis but not AKT or ERK.

**Conclusions:**

Ursolic acid-enriched KDCE enhances the antitumor activity of BCI, which could be mediated by VEGFR2 downregulation and subsequent suppression of angiogenesis. Therefore, combination therapy with S.t△ppGpp and KDCE is a potential cancer therapeutic strategy.

**Supplementary Information:**

The online version contains supplementary material available at 10.1186/s12906-022-03612-2.

## Background

Immunotherapy is the fourth-leading cancer therapy following surgery, radiotherapy, and chemotherapy [1, 2]. Immunological checkpoint therapies, such as CTLA-4, PD-L1, and PD-1, have been approved as a clinical cancer treatments [3], but also have limitations; “cold tumors” have decreased T cell infiltration and decreased antigen mutations, such as in pancreatic and ovarian cancers [4]. In addition, checkpoint therapy could cause autoimmune diseases by overactivating the immune system to attack normal tissues [5]. Bacteria-mediated cancer immunotherapy (BCI) was originally described over a century ago and has significant therapeutic advantages, including i) specific tumor targeting; ii) deep penetration of tumor tissue; iii) triggering robust antitumor immune stimulation; iv) low toxicity to normal tissues; v) lower cost than conventional immunotherapies [6].

Attenuated engineered *Salmonella* has been tested in recent clinical trials [7, 8]. *Salmonella*-mediated cancer immunotherapy profoundly affects the tumor microenvironment through several means: shifting macrophage phenotypes from M2 to M1 via toll-like receptors (TLRs) [9], initiating tumor cell apoptosis by inducing robust nitric oxide (NO) production in tumor cells [10], and inhibiting angiogenesis by downregulating vascular endothelial growth factor (VEGF) [11]. Angiogenesis has a very important role in tumor growth and metastasis. *Salmonella* combined with an antiangiogenic agents achieves improved therapeutic efficacy [12, 13].

*Ilex kudingcha* C. J Tseng is herbal tea in China, and bioactive compounds in this natural product have multiple therapeutic effects, including anticancer [14], anti-inflammatory [15], antidiabetic [16], and hypolipidaemic effects [17]. Ursolic acid is a pentacyclic triterpene acid and contributes to the anticancer activity of kudingcha [18]. Ko et al. reported that ursolic acid significantly inhibits cancer progression via phosphorylation of the ERK and AKT [19]. Prior studies also identified that ursolic acid nanoparticle-coated attenuated *Salmonella typhimurium* significantly suppresses tumor growth and metastasis [20].

The present study aimed to determine the combined effects of *Salmonella*-mediated cancer immunotherapy and ursolic acid-enriched kudingcha extracon antitumor activity in a mouse colon cancer model.

## Methods

### Plant material

*Ilex kudingcha* leaves were collected from Kudingcha Institute (Hainan University, Haikou, Hainan, China) and the state permissions were unnecessary to collect the sample. The plant material was identified by their morphological characteristics by Dr. Guomin Liu from the Kudingcha Institute, Hainan University. One voucher specimen (H. Y. Liang 60,355) was deposited at the Kudingcha Institute. Dried leaves were ground and passed through a sieve (24 mesh). Kudingcha powder (100 g) was boiled twice in water. The collected residue was extracted twice with 4 L 100% EtOH for 48 h, then subjected to ultrasound-assisted extraction at 50 °C for 30 min. The solvent was removed by rotary evaporation to yield a dry extract, which was dissolved in petroleum ether, evaporated, and freeze-dried to remove organic solvents. The petroleum ether fraction was used for further photochemical analysis and experiments.

### HPLC-PDA analysis

An HPLC-PDA system (Waters Corporation, Milford, MA, USA) consisting of a Waters 600 pump and a 996 PDA detector was used. Analyses were performed using a Waters Sun-Fire C18 column (4.6 × 150 mm, 5 μm). Chromatography conditions were as follows: MeOH: H_2_O, 90: 10 to 40: 60 for 40 min; MeOH: H_2_O, 40: 60 to 100% MeOH for 1 min; and 100% MeOH for 9 min. The flow rate was 0.1 mL/min; injection volume was 20 μL kudingcha extract (20 mg/mL in petroleum ether); and detection wavelengths were 220, 254, and 280 nm.

### Mouse colon cancer model and bacterial injection

BalB/c mice (male, 5–6 weeks old) were purchased from Guangdong Experimental Animal Center (Guangzhou, Guangdong, China). Experiments were supervised by the Animal Science and Technology Ethics Committee of Hainan University. Mice were anesthetized with either 2% isoflurane or ketamine (200 mg/kg). CT-26 cells (1 × 10^6^, ATCC) cultured in DMEM with 10% FBS (Gibco, USA) were implanted subcutaneously into the right flank to generate colon cancer xenografts. Tumor-bearing mice were randomly divided into four groups (*n* = 9/group) as follows: PBS, SLΔppGpp, KDCE, and SLΔppGpp + KDCE. KDCE groups received 1 g/kg KDCE daily via intragastric administration. When tumor volume reached 120–160 mm^3^, 1 × 10^7^ colony-forming units (CFU) SLΔppGpp bacteria were intravenously injected. When tumor volume reached ≥1500 mm^3^, mice were euthanized. Tumor volumes were measured and calculated using the following formula: (L × H × W)/2 (L: length; W: width; H: height).

Attenuated *Salmonella typhimurium*, S.t△ppGpp (defective in the synthesis of ppGpp (*RelA::cat*, *Spot::kan*)) carrying the luciferase gene *Lux* (S.t△ppGpp-lux; SHJ2168, 9] was kindly provided by J. J. Min (Institute for Molecular Imaging and Theranostics, Chonnam National University Hwasun Hospital, Jeonnam, Republic of Korea) and grown in Luria Bertani medium containing kanamycin (Sigma-Aldrich). Bacteria were stored in 25% glycerol stocks at − 80 °C.

### Bacteria counting and optical bioluminescence imaging

Tissues, including tumor, liver, lung, and spleen, were collected from mice. Ground tissue was transferred to agar petri dishes and incubated overnight at 37 °C. The bacterial number per gram of tissue was calculated by the formula: Y × 10^Z^ × (1 + X) × 10/X (CFU/g; X: tissue weight; Y: bacterial number on the petri dish; Z: dilution factor).

Tumor-bearing mice were injected through the tail vein with S.t△ppGpp Lux in 100 μL PBS to image bacterial bioluminescence imaging and divided into two treatment groups (*n* = 6/group): S.t△ppGpp Lux and S.t△ppGpp Lux + KDCE (intragastric administration 1 g/kg daily). D-luciferin (750 μg, Caliper, Hopkinton, MA, USA) was intraperitoneally injected, and bioluminescence imaging was then performed using an IVIS 100 (Caliper).

### H&E staining

Liver, spleen, kidney, and lung were removed from euthanized mice and fixed in 4% PFA solution for toxicity evaluation of KDCE + S.t△ppGpp. Paraffin sections (10 μM) were stained with a Hematoxylin and Eosin Staining Kit (C0105, Beyotime) according to the manufacturer’s protocol.

### Western blotting

Human Umbilical Vein Endothelial Cells (HUVECs, ATCC) were treated with KDCE (0, 40, 80, 100, 120, and 160 μg/ml) for 24 h. Protein lysates were separated by 10% SDS-PAGE and transferred to PVDF membranes (Merck, Darmstadt, Germany), which were incubated overnight at 4 °C with primary antibodies against VEGFR2 (sc-6251), p-ERK (sc-136,521), ERK1/2 (sc514302), AKT1/2/3 (sc56878), p-Akt (sc-293,125), and β-actin (sc-69,879) (Santa Cruz Biotechnology, Inc., Texas, USA). Membranes were then incubated with secondary antibodies. Protein bands were visualized using a chemiluminescence detection kit (ATTO, Tokyo, Japan) and semi-quantified using Image J Ω (Media Cybernetics, Maryland, USA).

### Statistical analyses

Statistical analyses were conducted using the GraphPad Prism 5.0, and *p* < 0.05 was considered statistically significant. Survival analysis was conducted using the Kaplan-Meier method and a log-rank test. All data are expressed as means ± SEM.

## Results

### KDCE Ursolic acid content

**Fig. 1:**
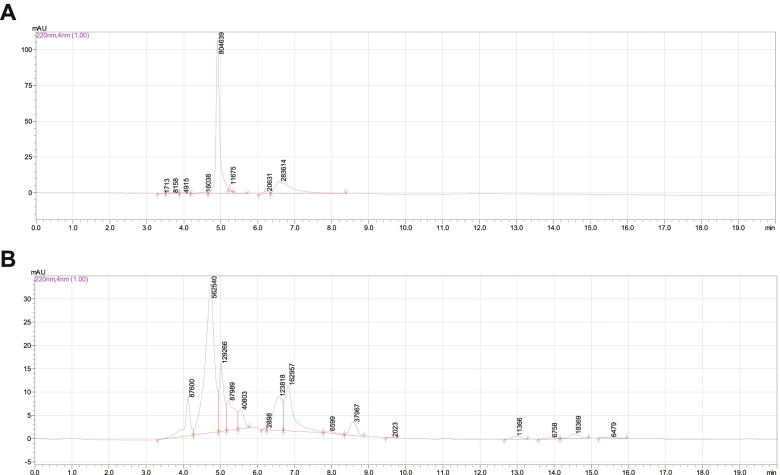
KDCE contains 134 mg/g of ursolic acid. HPLC chromatograms for ursolic acid standard solution (**A**) and kudingcha samples (**B**)

Leaves of *I. kudingcha* were collected from the Kudingcha Institute, Hainan University, Hainan province, China, in July 2020. Kudingcha powder (100 g) was extracted with boiled water, 100% EtOH, and petroleum ether in turn. The yield petroleum ether fraction (7.49 g) was used to detect the concentration of ursolic acid in KDCE using spectrophotometric analysis. Results indicated that KDCE contains 134 mg/g of ursolic acid using purified standards as calibrators at 220, 254, and 280 nm (Fig. 1).

### Effect of KDCE on anticancer activity of BCI

**Fig. 2:**
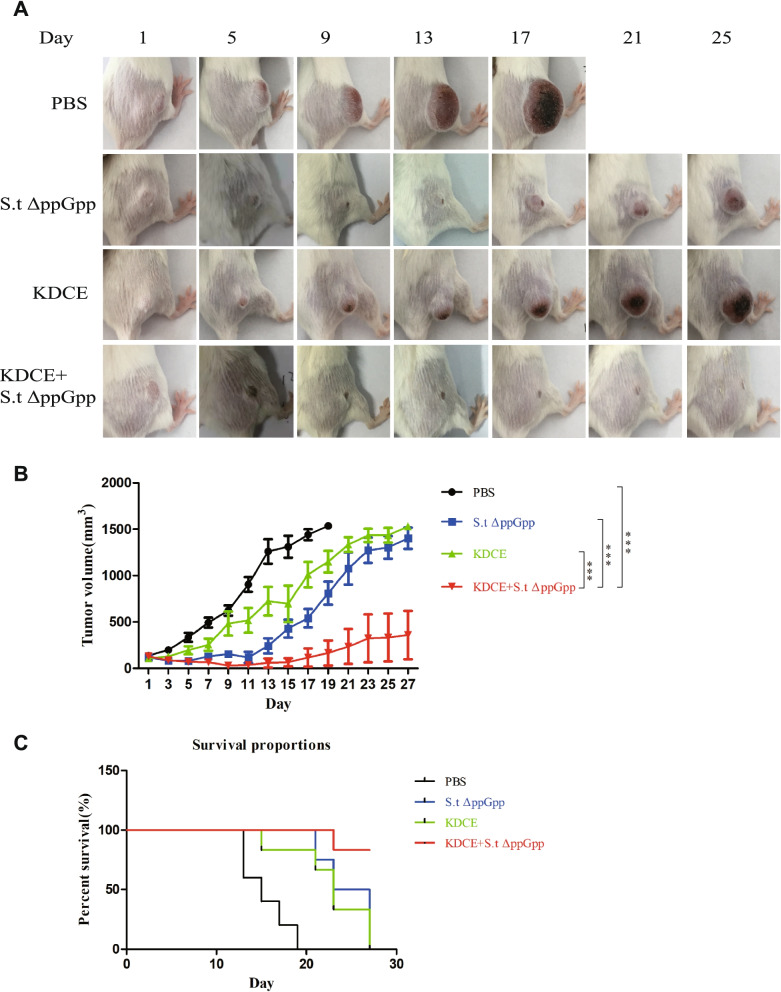
Effect of KDCE on S.t△ppGpp BCI. KDCE combined with SLΔppGpp show synergistic effect in suppressing tumor growth and increased survival rate. **A** Representative images of tumors for each group over time. **B** Tumor volume measurement (BalB/c mice, *n* = 9/group). **C** Kaplan-Meier survival curve. Statistical significance was calculated by comparison with PBS or S.t△ppGpp-alone groups (***p < 0.001)

To evaluate the effect of KDCE on the therapeutic efficacy of BCI, SLΔppGpp (3 × 10^7^ CFU) was injected into tumor-bearing mice via the tail vein on day 1, followed by intragastric administration of KDCE (1 g/kg) daily from day 2 to day 27. KDCE had antitumor activity, and when combined with SLΔppGpp had a strong synergistic effect in suppressing tumor growth (Fig. 2A and B) and increased survival rate (Fig. 2C).

### Effect of KDCE on tumor-colonizing bacteria

**Fig. 3:**
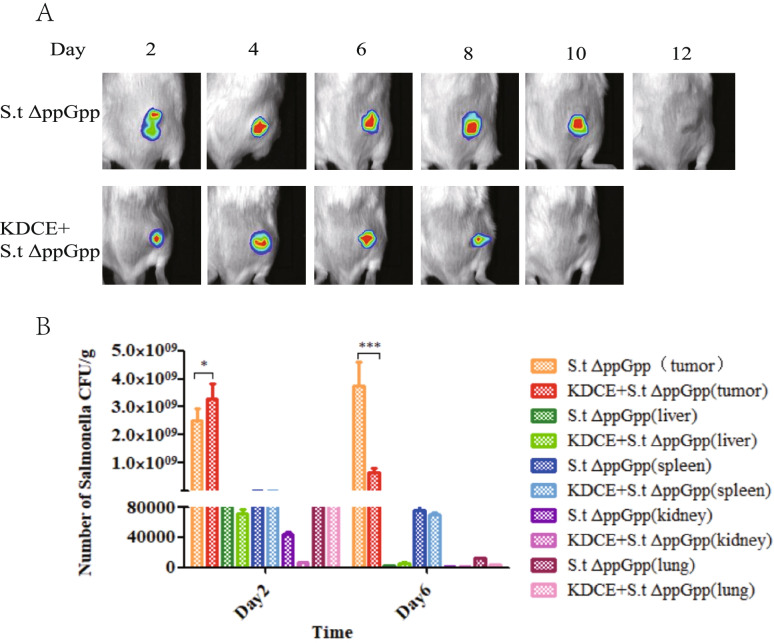
Effect of KDCE on tumor-colonizing bacteria. The survivor and number of tumor-colonizing bacteria in the SLΔppGpp plus KDCE group was lower than in the SLΔppGpp-only group (**A**) Non-invasive monitoring of bacterial bioluminescence for 12 days (BalB/c mice, *n* = 6/group). **B** Bacterial counts in isolated organs from tumor-bearing mice on days 2 and 6 (BalB/c mice, *n* = 6/group). Statistical significance was calculated by comparison with S.t△ppGpp-alone groups (*p < 0.05, ***p < 0.001)

To determine the effect of KDCE on tumor-colonizing bacteria, bacterial activity was measured by bioluminescence analysis, in which tumor-bearing mice were injected with SLΔppGpp-Lux. The bacterial tumor-colonizing time in the SLΔppGpp + KDCE group (8 days) was shorter than in the SLΔppGpp-only group (10 days) (Fig. 3A).

Subsequently, to determine bacterial distribution in major organs, we collected tumor, lung, liver, and spleen samples from bacteria-injected tumor-bearing mice on days 2 and 6. The tumor-colonizing bacterial number in the SLΔppGpp + KDCE group was higher on day 2 but lower on day 6 compared to the SLΔppGpp-only group (Fig. 3B).

### Toxicity evaluation of KDCE/BCI combination therapy

**Fig. 4:**
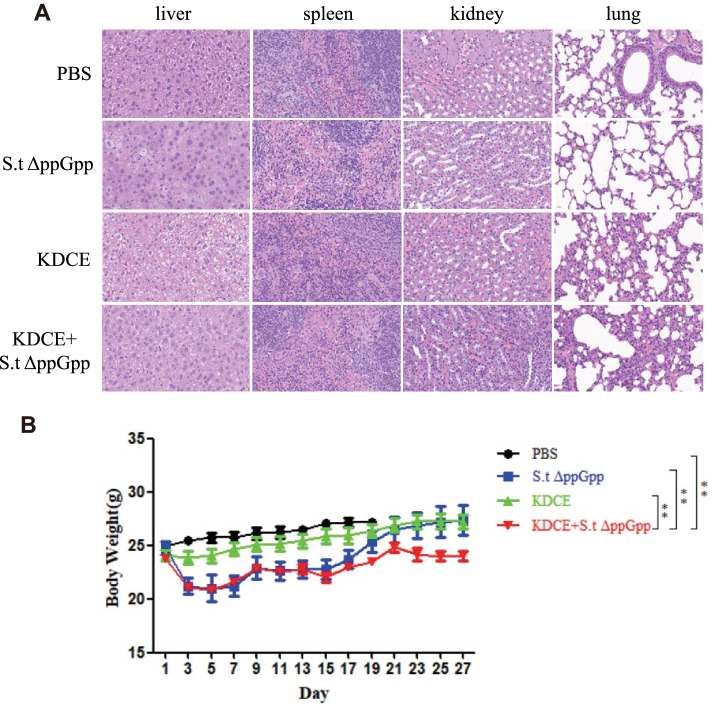
Toxicity evaluation of KDCE combined with S.t△ppGpp. **A** H&E staining for isolated organs of tumor-bearing mice on day 6 after BCI treatment (BalB/c mice, *n* = 6/group). No signs of steatosis, inflammatory infiltrate or fibrosis in the liver, spleen, kidney, and lung were observed in each group. **B** Body weight on day 27 (BalB/c mice, *n* = 6/group). Statistical significance was calculated by comparison with PBS or S.t△ppGpp-alone or KDCE groups (**p < 0.01)

To evaluate the safety of KDCE/BCI combination therapy, we collected lung, liver, and spleen samples from SLΔppGpp-injected tumor-bearing mice on day 6 after KDCE treatment for H&E staining. SLΔppGpp + KDCE did not induce toxicity in the liver, spleen, kidney, or lung as compared to other groups (Fig. 4A) but did decrease body weight (Fig. 4B).

### KDCE VEGFR2 downregulation

**Fig. 5:**
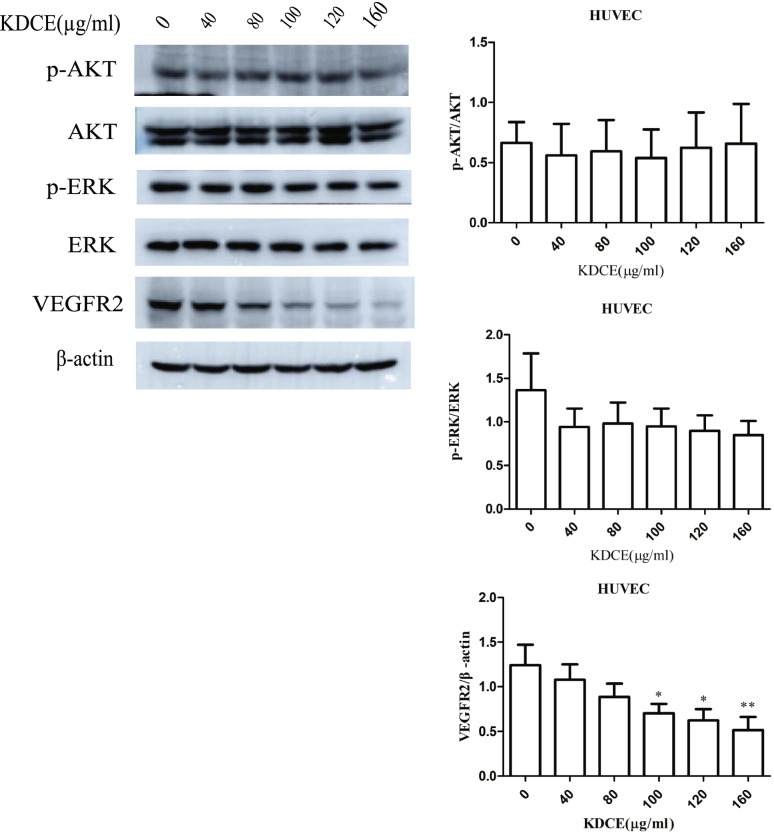
Effect of KDCE on protein expression of p-AKT, AKT, p-ERK, ERK, and VEGFR2. VEGFR2 was downregulated, but not p-AKT or p-ERK

To determine if the anticancer activity of KDCE was related to suppression of angiogenesis, we measured AKT and ERK phosphorylation and VEGFR2 levels using western blotting in HUVECs. KDCE significantly downregulated VEGFR2 in a dose-dependent manner, but did not affect AKT or ERK phosphorylation (Fig. 5).

## Discussions

The delivery and efficiency of the drug are improved by nano-encapsulating, but FDA approval and commercialization of nanomaterial still is a major challenge [21–23]. We previously observed remarkable tumor-targeting and therapeutic effects using hydrocypropyl-β-cyclodextrin (HPCD)-encapsulated ursolic acid coating the surface of S.t△ppGppand by amantadine (AMA) [20]. However, considering ursolic acid-enriched KDCE is easier to access in a clinical trial than nano-encapsulated ursolic acid, we determined the tumor therapeutic effect of KDCE combined with S.t△ppGpp.

*Salmonella*-mediated cancer immunotherapy stimulates immune activation and inhibits angiogenesis by downregulating VEGF [11]; Kudingcha [24] and ursolic acid induces cancer cell apoptosis [25]. Here, we demonstrated that ursolic acid-enriched KDCE enhances the antitumor activity of S.t ΔppGpp-mediated cancer immunotherapy, but does not increase bacterial tumor-colonizing time or number. We postulated that ursolic acid-enriched KDCE could decrease tumor volume by downregulating VEGFR2, limiting bacterial colonization. Several prior studies reported that VEGFR2 downregulation plays important role in suppressing tumor angiogenesis, which is related to suppression of ERK [26–28] and AKT [29–31]. However, our findings suggest that KDCE-induced VEGFR2 downregulation is not related to deactivation of the AKT or ERK pathways. The prior studies reported that VEGFR2 downregulation must be due to decreased VEGF synthesis, which auto-regulates its receptor, VEGFR2. In addition, the phosphorylation FAK and Matrix metalloproteinases 9 (MMP9) and Signal transducer and activator of transcription 3 (STAT3) interact with VEGF signaling [32, 33]. Hence, we speculated that perhaps KDCE-induced VEGFR2 downregulation is associated with FAK/MMP9/STAT3 axis.

The current research has been focused on bacteria-mediated gene therapy of delivering therapeutic drugs for enhancing the efficacy of BCI [34–36], but our investigation indicated that a combination of BCI and nature products having anti-angiogenesis function is also an alternative approach.

## Conclusions

The present study demonstrated that ursolic acid-enriched KDCE enhances the antitumor activity of BCI, which could be mediated by VEGFR2 downregulation and subsequent suppression of angiogenesis. Combined BCI and KDCE treatment is a potential modality for cancer therapy.

## Supplementary Information

**Additional file 1.**

## Data Availability

All data generated or analyzed during this study are included in this published article and its supplementary information files.

## Abbreviations

BCI: Bacteria-mediated cancer immunotherapy
KDCE: Kudingcha extract
TLRs: Toll-like receptors
VEGF: Vascular endothelial growth factor
VEGFR2: Vascular endothelial growth factor receptor 2
S.t△ppGpp: Attenuated *Salmonella typhimurium*
ERK: Extracellular Regulated protein Kinases
AKT: Protein Kinase B
HPCD: Hydrocypropyl-β-cyclodextrin
AMA: Amantadine
MMP9: Matrix metalloproteinases 9)
STAT3: Signal transducer and activator of transcription 3
