# Supplementary figures and images for: Ursolic acid-enriched kudingcha extract enhances the antitumor activity of bacteria-mediated cancer immunotherapy
Source: BMC Complement Med Ther. 2022 May 4;22:123. doi: 10.1186/s12906-022-03612-2 (PMC9066986; doi:10.1186/s12906-022-03612-2)

Supplementary X

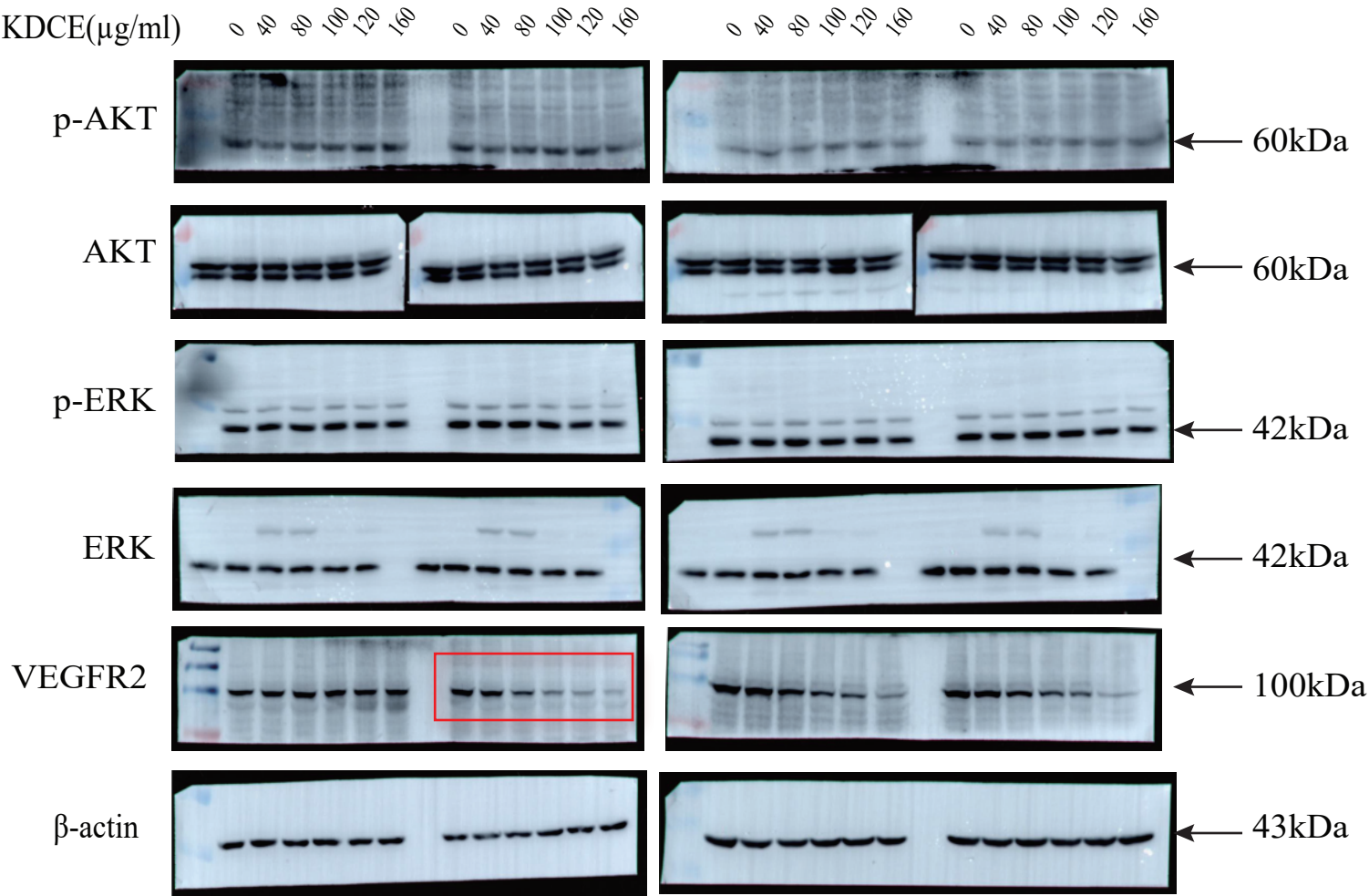

Supplement: Supplementary file 1 — Additional file 1. [file 12906_2022_3612_MOESM1_ESM.pdf]
